# Measurement properties of the PROMIS-29 profile v2.1 in a Norwegian rehabilitation context

**DOI:** 10.1186/s41687-025-00929-7

**Published:** 2025-07-31

**Authors:** Stein Arne Rimehaug, Rikke Helene Moe, Hanne Dagfinrud, Felix Fischer, Thomas Johansen, Ingvild Kjeken, Mari Klokkerud, Hanne Ludt Fossmo, Anne Dorte Lyken, Tarja Rajalahti Kvalheim, Silje Soldal, Anne-Lene Sand-Svartrud, on behalf of The RehabNytte Consortium

**Affiliations:** 1 Regional Rehabilitation Knowledge Center (RKR), Sunnaas Hospital, Oslo, Norway; 2 Health Services Research and Innovation Unit, Center for Treatment of Rheumatic and Musculoskeletal Diseases (REMEDY), Diakonhjemmet Hospital, Oslo, Norway; 3 Charité – Universitätsmedizin Berlin, Freie Universität Berlin and Humboldt Universität zu Berlin, Medizinische Klinik mit Schwerpunkt für Psychosomatik, Center for Patient-Centered Outcomes Research, Berlin, Germany; 4 Hernes Occupational Rehabilitation Center, Hernes, Norway; 5 Department of Rehabilitation Science and Health Technology, Faculty of Health Science, OsloMet University, Oslo, Norway; 6 Vikersund Rehabilitation Center, Vikersund, Norway; 7 Sørlandet Rehabilitation Center, Eiken, Norway; 8 Red Cross Haugland Rehabilitation Center, Flekke, Norway; 9 Ravneberghaugen Rehabilitation Center, Hagavik, Norway

**Keywords:** Patient-reported outcome measures, PROMIS, Psychometrics, Rehabilitation, Outcome measures, Quality of life

## Abstract

**Introduction:**

Psychometric properties of the Patient-Reported Outcomes Measurement Information System^®^ Profile 29 (PROMIS-29) Norwegian version has previously been examined in a general population. This multicenter study aimed to examine the internal consistency, construct validity, responsiveness, score distribution and floor/ceiling effects of PROMIS-29 v2.1 in a Norwegian rehabilitation context.

**Methods:**

Patients receiving rehabilitation services participating in a longitudinal cohort study answered PROMIS-29 at baseline and at 3-month follow-up. Internal consistency was assessed by Cronbach’s alpha and McDonald’s omega. Construct validity was examined through hypothesis testing, using EQ-5D-5L as comparator measure. Hypotheses for correlations of change scores for both questionnaires were tested as an expression of responsiveness. Score distribution and floor/ceiling effects were examined with histograms and descriptive statistics.

**Results:**

A total of 828 patients with a mean age of 54.3 years were included for analysis. The internal consistency for each PROMIS-29 domain was confirmed, with alpha and omega values exceeding the threshold of ≥ 0.70. Regarding correlations between PROMIS-29 and EQ-5D-5L, 34 out of 40 hypotheses were confirmed for construct validity and 19 out of 24 for responsiveness, both meeting our a priori criterion of ≥ 75% confirmed hypotheses. There was no floor effect of any PROMIS-29 domain in our sample, and ceiling effect only for anxiety and depression domain scores. Still, this confirmed the applicability of PROMIS-29 in a rehabilitation context.

**Conclusion:**

The Norwegian PROMIS-29 has sufficient internal consistency, construct validity and responsiveness for use as an outcome measure for health status and health-related quality of life in rehabilitation.

**Trial registration number:**

ClinicalTrials.gov NCT03764982 RehabNytte study, registered 2018-12-04.

**Supplementary Information:**

The online version contains supplementary material available at 10.1186/s41687-025-00929-7.

## Background

One in three people worldwide will need rehabilitation during the course of illness or injury, often due to conditions like musculoskeletal, neurological or cardiovascular diseases [1]. Some need interventions from multidisciplinary teams in secondary healthcare [2]. In Norway, rehabilitation is provided in public hospitals and publicly funded private rehabilitation centres, with follow-up by local municipal teams when needed [2].

Rehabilitation services help patients regain or maintain optimal function, meaningful activities, and participation in social and work life through collaborative processes and tailored interventions [3, 4]. These interventions target the impact of disease, comorbidities, or treatment side effects on self-perceived functioning and well-being [5, 6]. Accordingly, health-related quality of life (HRQoL) is crucial in rehabilitation, reflecting patients’ physical, mental, and social experiences [5, 6]. Patient-reported outcome measures, like the increasingly used Patient-Reported Outcomes Measurement Information System^®^ (PROMIS), provide valuable insights into how medical conditions and interventions affect HRQoL, enabling patient-centered care [6–9].

The PROMIS-29 Health Profile v2.1 (PROMIS-29) is a 29-item questionnaire developed in the United States to standardize HRQoL in research and clinical practice [9, 10]. It covers seven health domains: Physical Function, Depression, Anxiety, Fatigue, Sleep Disturbance, Ability to Participate in Social Roles and Activities (Social Participation), and Pain (Interference and Intensity). PROMIS-29 is intended for use both for patients with chronic diseases and the general population. The questionnaire has been translated into many languages and evaluated for its measurement properties across various populations [9–17]. The Norwegian version of PROMIS-29 has shown sufficient reliability and structural, convergent and discriminant validity in the general population in studies applying cross-sectional designs [18, 19]. However, further analyses are needed to assess these properties, including responsiveness to change in a longitudinal context, as well as any floor or ceiling effects, in a rehabilitation setting.

Thus, the aim of this study was to assess internal consistency, construct validity and responsiveness of PROMIS-29 v2.1 in a multicentre rehabilitation setting. Additionally, we aimed to examine potential floor and ceiling effects in a diverse sample of adults with chronic diseases requiring multidisciplinary rehabilitation in secondary healthcare.

## Methods

### Study design and clinical setting

The RehabNytte Project was a large, longitudinal cohort study following patients referred for rehabilitation in one of 17 rehabilitation centres from January 2019 to March 2020 [20]. The centres were all private institutions which were contracted to the public, secondary healthcare system in Norway, and part of the VIRKE Research and Development Network. This network established a common digital database, The RehabNytte Cohort, for monitoring the patients’ progress on PROMs and their overall benefits of rehabilitation services. The patients completed a set of PROMs at five different time points during a year. The set of PROMs included the assessment of work ability, pain, acceptability, change in health status, health-related quality of life, patient-specific rehabilitation goals, and broader aspects of functioning. The latter was assessed by the PROMIS-29 [20].

The current study was a planned and independent study within the RehabNytte Project, aiming to examine the selected measurement properties of PROMIS-29. At one RehabNytte-centre, the PROMIS-29 was not included in their set of PROMs. At another centre, their one-week-course was not comparable to the rehabilitation programs among the other centres. Therefore, the current study included 15 out of 17 RehabNytte centres.

Eligible patients were referred to one of the 15 centres primarily for somatic illness, while a smaller group was referred due to a complex interplay of somatic and mental health issues. Other inclusion criteria were age ≥ 18 years, able to read and understand questionnaires in Norwegian, and access to a smartphone, tablet or equivalent devices necessary to complete digital data collection. The exclusion criteria were current participation in other research projects, severe cognitive impairment or mental illness influencing their ability to complete the patient-reported outcome measures during their rehabilitation stay and in the subsequent 12 months follow-up period [20].

At each centre, patients followed a multidisciplinary rehabilitation program provided by at least four professionals, such as nurses, physiotherapists, occupational therapists, and medical doctors. Some teams also included a social worker, a nutritionist/dietitian, sports educator, and/or a psychologist. Most centres delivered inpatient stays for 2–4 weeks. Some (6/15) provided outpatient rehabilitation in addition, for patients living in short distance to the institution.

Individual and group-based sessions included activities of daily living, physical activity and exercise, as well as patient education on coping (fatigue, pain, sleep, and/or stress), and healthy lifestyle changes (weight loss/control, and/or smoke cessation). When relevant, the sessions also addressed family and social relationships, work and work adaptations, social services and rights. The programs were tailored to patients’ needs and agreed goals set in collaboration between the individual patient and the rehabilitation team.

Patient research partners and clinician representatives were actively involved from developing to implementing the project plans [20]. The study was approved by the data protection officer at Diakonhjemmet Hospital (DS-00040, dated 17.10.2018), and registered in ClinicalTrials.gov (NCT03764982) [20]. Further ethical approval was not required (2018/1645/ the Norwegian Regional Committee for Medical Research Ethics, South-East A). Written informed consent from patients was obtained upon inclusion. Testing and reporting of measurement properties followed the COSMIN checklist and guidelines [21, 22].

### Data collection and measurements

As part of the larger RehabNytte project, data were collected digitally, using a secure system (CheckWare) approved in accordance with the EU general data protection regulations. Patients completed items on demographics as listed in Table 1, as well as a set of PROMs at admission to rehabilitation (T1, baseline), discharge (T2) and 3, 6 and 12 months after admission (T3-6). In the current study, we utilized data from the PROMIS-29 and EQ-5D-5L questionnaires, collected at T1 (completed at the rehabilitation centre) and T3 (completed at home). At each time point, patients received an automated email and a text message on their phones containing a link to the data collection system. Non-responders were sent a reminder via email and text message one week later. The interval between T1 and T3 was deemed the most appropriate timeframe for evaluating responsiveness [21]. The RehabNytte cohort was limited to participants who provided responses to at least one item on both questionnaires at each time point.

### The PROMIS-29 profile

Each of the health domains is assessed with four questions (items), except for Pain Intensity, which is measured on an 11-point numeric rating scale from 0 (“no pain”) to 10 (“worst imaginable pain” [10]. Each question has a response scale ranging from 1 to 5 (raw score); “Never”, “Without any difficulty”, or “Not at all” = 1, “Rarely”, “With a little difficulty”, or “A little bit” = 2, “Sometimes”, “With some difficulty” or “Somewhat” = 3, “Often”, “With much difficulty”, or “Quite a bit” = 4, and “Always”, “Unable to do”, or “Very much” = 5. A 7-day recall period is used for all domains except Physical Function and Social Participation, which assess current abilities. The total raw score or item response pattern for each domain should be converted to a standardized T-score. We used previously estimated item parameters, which were calibrated during PROMIS development, using the PROMIS wave 1 data [23] and the Graded Response IRT model to generate T-scores from the item response patterns for each domain. T-scores represent a mean of 50 and a standard deviation of 10 compared against the United States general population [10]. For the domains Physical Function and Social Participation, higher T-scores indicate better health, while for Anxiety, Depression, Fatigue, Sleep Disturbance and Pain Interference, higher T-scores indicate poorer health [10].

### The EQ-5D-5L questionnaire

The EQ-5D questionnaires are standardized measures of health status, developed by the international EuroQol Group to provide a generic tool for clinical and economic evaluations [24, 25]. Using the EQ-5D-5L [25], patients respond to five health dimensions: Mobility, Self-Care, Usual Activities, Pain/Discomfort, and Anxiety/Depression. Each dimension is assessed with a single item, using response options ranging from 1 to 5 (raw score) to indicate the severity level: “no problems” or “no symptoms” = 1, “slight problems / symptoms” = 2, “moderate problems / symptoms” = 3, “severe problems / symptoms” = 4, and “unable” or “extreme symptoms” = 5, [25–27]. In addition, patients rate their overall current health state on a 100 mm visual analogue scale (EQ VAS), with 0 indicating “The worst health you can imagine” and 100 indicating “The best health you can imagine”. The recall period is set to “today” [28]. The EQ-5D-5L is widely used for research and clinical practice. It has demonstrated validity and reliability across diverse countries, populations and health settings, including rehabilitation and chronic diseases [29].

### Floor and ceiling effects

We analyzed the distribution of the PROMIS-29 scores using the T-scores per domain. The results are presented by mean and standard deviation, as well as by histograms for visual comparisons. Further, we analyzed the raw scores and calculated the percentage of patients who reported the maximum or minimum scores at T1. A clustering of scores (≥ 15%) at the upper or lower end of the scale may indicate floor and ceiling effects [30, 31]. Missing values ≤ 5% was considered sufficient [31]. At the domain level we used PROMIS-29 per domain raw scores, ranging between 4 and 20. At the item level, raw scores ranged from 1 to 5. A floor effect was defined as reporting at the end of worst health status, in terms of lowest possible domain score for Physical Function and Social Participation, and highest possible domain score for Anxiety, Depression, Fatigue, Sleep Disturbance, Pain Interference, and Pain Intensity. The results addressing missing values, floor and ceiling effects are presented as percentages.

### Internal consistency

In this study, internal consistency refers to the extent to which the four items within each domain in the PROMIS-29 Profile are correlated with one another, demonstrating that they consistently measure the same underlying aspect of HRQoL [21, 30]. We utilized item raw scores measured at T1. To ensure a robust evaluation of internal consistency, we used both Cronbach’s alpha (α) and McDonald’s omega (ω) [22, 31]. Internal consistency was regarded sufficient if the estimate was ≥ 0.70 for each domain [32], and excellent at 0.90 [33].

### Construct validity

Construct validity refers to the degree to which the instrument provides scores that align with established knowledge about the underlying construct it aims to measure [21]. We investigated the validity by testing hypotheses about expected relationships with another outcome measure addressing the HRQoL concept [30]. Therefore, we assessed construct validity by testing predefined hypotheses about the expected relationships of scores of the PROMIS-29 Profile with scores of the EQ-5D-5L questionnaire [21, 30]. Our rationale for the hypotheses derived from the assumed conceptual overlap between the two questionnaires, as both are designed to measure patients’ self-perceived HRQoL [34]. The hypotheses were based on the presence of both overlap and divergence in the dimensions covered by PROMIS-29 and EQ-5D-5L, existing evidence regarding correlations between their scores [18, 35], and by clinical and scientific expertise within the research group. Preliminary hypotheses developed by three of the authors (SAR, RHM, ALSS) were discussed and refined until final versions were established by a larger research group (SAR, IK, HD, RHM, ALSS). Our hypothesized classification of PROMIS-29 domains with EQ-5D-5L dimensions, based on their degree of similarity, is presented together with the results in Table 4, as well as in Additional file 1 for more details. We established an 8 × 5 mapping table, in which the PROMIS-29 domains (*n* = 7) plus the Pain Intensity Scale comprised the rows and the EQ-5D-5L dimensions (*n* = 5) comprised the columns. Having 40 hypotheses for evaluation of the construct validity, 7 cells reflected the same constructs, 13 cells reflected largely related but dissimilar constructs, 15 cells reflected moderately related but dissimilar constructs, and 5 cells reflected weakly related or unrelated constructs.

The criteria for expected levels of correlation adhered to those used in a prior study conducted within the general population in Norway [18], as this formed a central part of our rationale for the hypotheses. Thus, correlations were expected to be ≥ 0.60 for scores assessing the same construct, < 0.60 and ≥ 0.30 for scores assessing largely related but dissimilar constructs, < 0.50 and ≥ 0.20 for scores assessing moderately related but dissimilar constructs, and < 0.30 for scores assessing weakly related or unrelated constructs [36]. For hypothesis testing, we compared the T-score for each PROMIS-29 domain and the EQ-5D-5L item scores, except for the PROMIS Pain Intensity domain, where we used the numeric rating scale score.

### Responsiveness

Responsiveness is regarded as an indicator of longitudinal validity [30]. We assessed responsiveness by testing hypotheses regarding expected correlations between changes (∆) in scores of the PROMIS-29 Profile and changes in scores of EQ-5D-5L [21, 30]. We expected patients’ HRQoL to either improve, deteriorate, or remain stable from T1 to T3, due to effectiveness of the rehabilitation interventions, the adoption of new self-management strategies, or the fluctuating or progressive nature of chronic diseases or comorbidities.

In parallel with developing validity hypotheses, we (SAR, RHM, ALSS) drafted preliminary responsiveness hypotheses. Given the limited evidence regarding the relationship between PROMIS-29 and EQ-5D change scores [34], we relied most on expert consensus within the research group (SAR, IK, HD, RHM, ALSS). Additionally, we used the mapping table and the results from the construct validity testing to finalize the pre-defined responsiveness hypotheses. We expected the correlations to be ≥ 0.50 for changes in pairs of domains/dimensions measuring similar constructs, < 0.50 and > 0.30 for pairs measuring related but dissimilar constructs, and < 0.30 for pairs measuring unrelated constructs [37]. Additional hypotheses addressed the relative correlations of change [30, 37]. In short, we developed 24 hypotheses; 10 addressing relative correlations and 14 addressing expected magnitude of the correlations. These are presented together with the results, in Table 3. Details regarding the rationale for the responsiveness hypotheses are given in Additional File 2. For the hypothesis testing, we compared changes in the T-score for each PROMIS-29 domain and changes in the EQ-5D-5L item scores, except for the PROMIS Pain Intensity domain, where we used changes in the numeric rating scale score.

The correlation analyses were conducted using the Spearman’s rho coefficient to assess both responsiveness and validity. The correlation values are presented as absolute values. Validity and responsiveness were deemed sufficient if at least 75% of the predefined hypotheses were confirmed [32, 37].

Statistical analyses were performed using SPSS Statistics v.29 and R v.4.3.1.

## Results

**Fig. 1:**
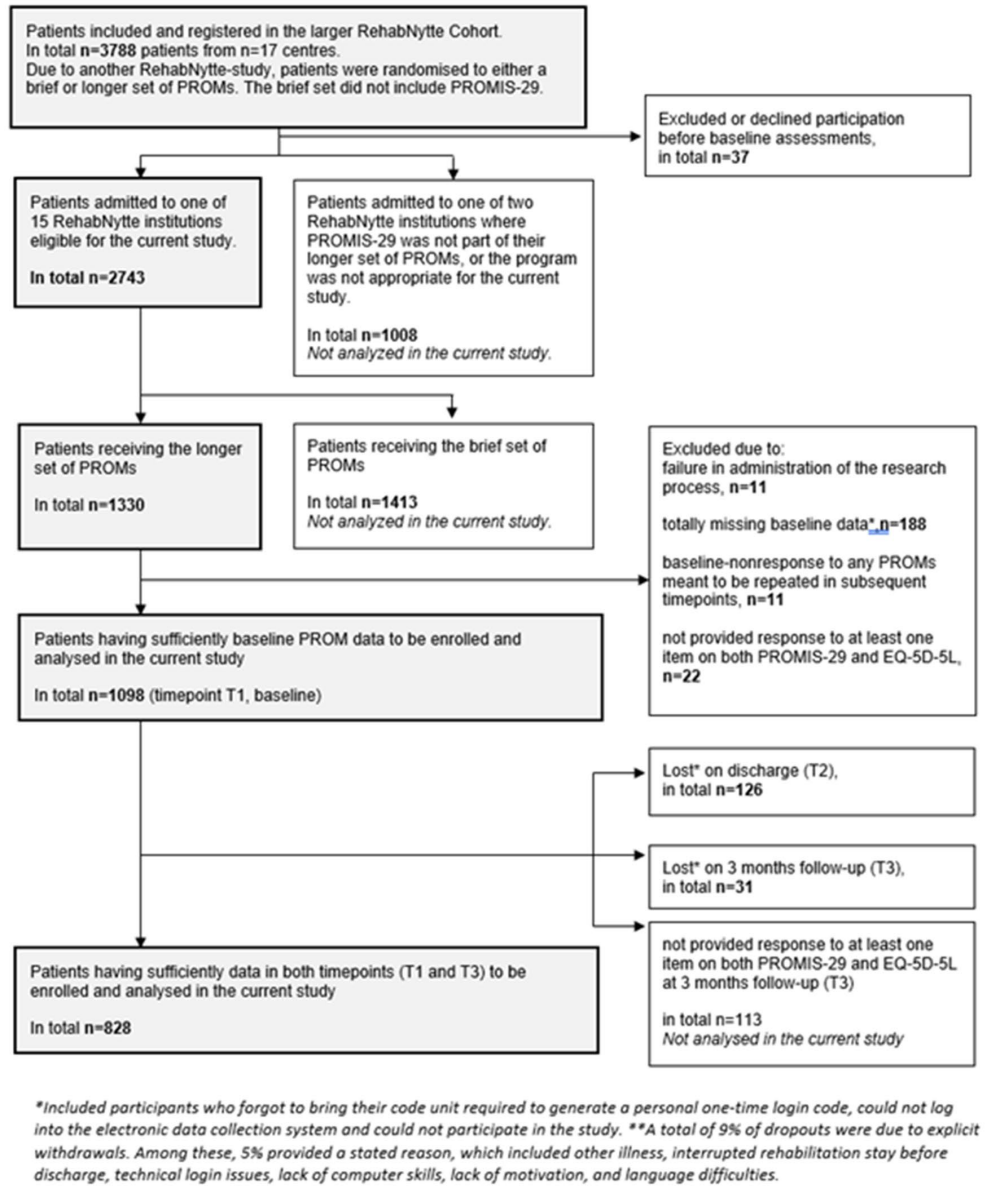
Flow diagram illustrating the selection of participants included in the current study

A total of 1098 patients completed one or more items of both PROMIS-29 and EQ-5D-5L at T1. The loss to follow up from T1 to T3 was 270/1098 participants (24.6%). A total of 828 patients, who also completed one or more items of both questionnaires at T3, formed our study sample (Fig. 1). The sample consisted of patients from all 15 participating centres, between 11 and 153 patients from each site. The majority (51.7%) had rheumatic or musculoskeletal diseases. The mean age was 54.3 years, 67.8% were female and 43.5% had higher education (Table 1). Demographic and clinical variables of the 270 respondents with incomplete or missing follow-up data were compared. Comorbidity (2.0 vs. 2.4 comorbidities – p-value < 0.01) and foreign native tongue respondents (8.9% vs. 5.2% - p-value < 0.01) were found to be significantly different, in addition to some differences in centre allocations. More details are given in Additional file 4.

**Table 1:** Baseline demographics of patients and their allocation across rehabilitation centres

Variables	Study sample (*n* = 828)
Age^a^, years, mean (SD)	54.3 (13.8)
Sex^a^, female, n (%)	561 (67.8)
Diagnosis^b^, n (%)	
Rheumatic or musculoskeletal diseases	428 (51.7)
Neurological disease	125 (15.1)
Lifestyle disease, overweight	93 (11.2)
Cancer	54 (6.5)
Sensory impairment	44 (5.4)
Cardiovascular disease	44 (5.4)
Mental disease	4 (0.5)
Other disease	36 (4.3)
Rehabilitation institution, n (%)	
Centre 1	43 (5.2)
Centre 2	96 (11.6)
Centre 3	28 (3.4)
Centre 4	58 (7.0)
Centre 5	35 (4.2)
Centre 6	153 (18.5)
Centre 7	94 (11.4)
Centre 8	13 (1.6)
Centre 9	16 (1.9)
Centre 10	65 (7.9)
Centre 11	11 (1.3)
Centre 12	23 (2.8)
Centre 13	44 (5.3)
Centre 14	104 (12.6)
Centre 15	45 (5.4)
**Patient-reported data**	
Comorbidities^c^ n, median (min, max)	2.4 (1, 10)
Body mass index kg/m^2^, mean (SD)	30.1 (7.0)
Smoking and/or snuff use, n (%)	201 (24.3)
Education > 12 years, n (%)	360 (43.5)
Paid work (currently, full or part time), n (%)	445 (54.0)
Recipients of social security benefits, n (%)	678 (81.9)
Language (native tongue), n (%)	
Norwegian, Swedish, or Danish	785 (94.8)
Other languages	43 (5.2)
Civil status, n (%)	
Married / cohabitant	514 (62.1)
Single	311 (37.6)
Caregiver for child(ren)/others in or outside home, n (%)	352 (42.5)
Annual gross income in the household > 600 000 NOK, n (%)	382 (46.0)

^a^Data collected from the national identification number. ^b^Clinician-reported data, mandatory, ^c^Self-reported, using a 19-tiem comorbidity checklist

### Baseline score distribution and floor/ceiling effects

**Fig. 2:**
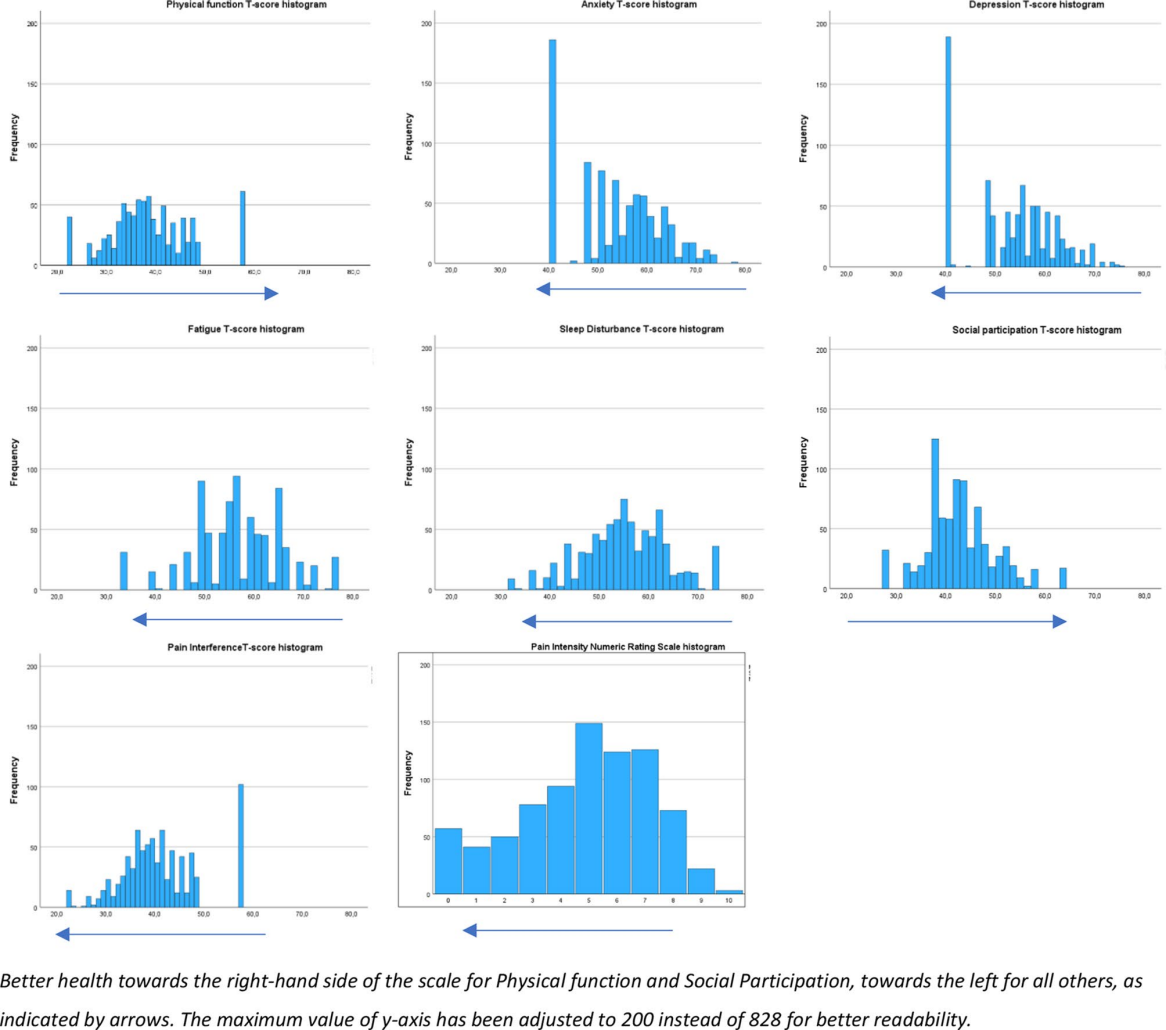
Distribution histograms for PROMIS-29 per domain T-scores at baseline

The PROMIS-29 mean scores at T1 deviated 3.4 to 11.3 T-score points from the United States general population reference of “50”, indicating that these patients on average had mild to moderate problems with Physical function, Fatigue, Social participation and Pain, and to a lesser extent with Sleep, Anxiety and Depression (Table 2). The histograms (Fig. 2) showed nearly normally distributed T-scores, but with some scores clustered at the lowest and/or highest possible score. T1-T3 mean change scores indicate a modest, but statistically significant improvement on a group level in all PROMIS domains (Table 2, details in Additional file 3).

**Table 2:** Distribution of PROMIS-29 per domain T-scores and change scores in the period from admission (T1) to the first follow-up measurement at home (T3)

PROMIS-29 domain (min-max possible score range)	T-score mean (SD) at T1	T-score mean (SD) at T3	Mean change (SD) in T-scores between T1 and T3
Physical function (22.5–57.0)	38.7 (8.1)	41.0 (8.0)	2.4 (6.6)
Anxiety (40.3–81.4)	53.4 (9.1)	52.1 (9.1)	-1.3 (7.3)
Depression (41.0–79.3)	53.4 (8.6)	52.7 (8.8)	-0.7 (7.6)
Fatigue (33.7–75.8)	56.6 (9.1)	53.6 (10.0)	-3.0 (7.8)
Sleep disturbance (32.0–73.3)	55.1 (8.5)	53.0 (8.3)	-2.1 (7.5)
Social Participation (27.5–64.2)	42.7 (7.2)	44.9 (8.1)	2.2 (6.7)
Pain Interference (41.6–75.6)	60.1 (8.7)	58.0 (8.9)	-2.1 (7.2)
	NRS-score mean (SD) at T1	NRS-score mean (SD) at T3	Change (SD) in NRS-scores between T1 and T3
Pain Intensity (NRS, 0–10)	4.8 (2.4)	4.3 (2.4)	-0.5 (2.0)

SD: standard deviation; T1: admission to rehabilitation; T3: three months after admission; NRS: numeric rating scale. Better health = positive T-score change for physical function and social participation, and negative change for all others

The proportion of missing (%) was well below 5% at both the item and domain levels (Table 3). No floor effects of ≥ 15% were observed in any of the PROMIS-29 domains in this sample, indicating that scores did not cluster substantially at the worst end of the health status scales. There was, however, a ceiling effect for PROMIS-29 Anxiety (22.5%) and Depression (22.8%), meaning about one fifth of the patients were at the best-health-status end of the scales for these domains (Table 3). There was a clustering of item scores (≥ 15%) at either end of the scale in four items related to Physical Function and two Sleep Disturbance items, without causing any floor/ceiling effect at the domain level for these domains. Results on item-level are provided in Additional File 3.

**Table 3:** Proportion of missing, floor and ceiling effects* by PROMIS-29 domain

PROMIS-29 domain	Missing (%)	Floor (%) (= worst health)	Ceiling (%) (= best health)
Physical Function	0.2	4.8	7.4
Anxiety	0.4	0.0	**22.5**
Depression	0.4	0.0	**22.8**
Fatigue	0.4	3.3	3.7
Sleep disturbance	0.4	4.3	1.1
Social Participation	0.4	3.9	2.1
Pain Interference	0.8	7.4	10.1
Pain Intensity	1.3	0.4	6.9

*Percentage per domain raw scores at “4” or “20”. Floor: worst health status, regardless of the scoring direction of the domain. Thus, low Physical Function and Social Participation scores, and high Anxiety, Depression, Fatigue, Sleep Disturbance and Pain scores were considered floor effects. Floor/ceiling effects at (≥ 15) at the domain level are marked with **bold numbers**

### Internal consistency

At T1, the values of both Cronbach’s Alpha and McDonald’s Omega fell within the range of sufficient internal consistency (≥ 0.70), and most PROMIS-29 domains were in the excellent range. The Cronbach’s Alpha values for each domain ranged between 0.85 and 0.96, and McDonald’s Omega total ranged between 0.88 and 0.95. In detail, the values for Alpha (Omega) were for Physical Function 0.91 (0.93), Anxiety 0.89 (0.92), Depression 0.89 (0.89), Fatigue 0.94 (0.93), Sleep Disturbance 0.85 (0.88), Social Participation 0.90 (0.91), and Pain Interference 0.96 (0.95).

### Construct validity

In total, 34 out of 40 hypotheses (85.0%) for correlations between PROMIS-29 domains and EQ-5D-5L dimensions were confirmed. All seven hypotheses for baseline correlations between pairs of *the same construct* were confirmed, as well as 11 out of 13 for *largely related but dissimilar constructs*, 11 out of 15 for *moderately related but dissimilar constructs*, and all five hypotheses targeting *weakly related or unrelated constructs* (Table 4).

**Table 4:** Expected similarity between PROMIS-29 domains and EQ-5D-5L dimensions, and correlation* results

Color coding: A priori expected correlation strengths are identified by background shading:	Weakly related or unrelated constructs < 0.30	Moderately related but dissimilar constructs < 0.50 and ≥ 0.20	Largely related but dissimilar constructs < 0.60 and ≥ 0.30	The same construct ≥ 0.60	
PROMIS-29 Domains:	EQ-5D-5L Mobility	EQ-5D-5L Self-Care	EQ-5D-5L Usual Activities	EQ-5D-5L Pain/ Discomfort	EQ-5D-5L Anxiety/ Depression
Physical function	**-0.76 ** [-0.78, -0.73]	**-0.57** [-0.60, -0.53]	**-0.67** [-0.70, -0.64]	**-0.47** [-0.51, -0.42]	**-0.04** [-0.09, 0.02]
Anxiety	**0.01** [-0.05, 0.06]	**0.05** [0.00, 0.10]	0.16 [0.11, 0.21]	**0.24** [0.19, 0.29]	**0.75 ** [0.73, 0.77]
Depression	0.11 [0.06, 0.16]	0.13 [0.08, 0.19]	**0.24** [0.19, 0.29]	**0.30** [0.25, 0.34]	**0.70** [0.67, 0.73]
Fatigue	**0.20** [0.15, 0.25]	**0.22** [0.17, 0.27]	**0.46** [0.41, 0.50]	**0.45** [0.41, 0.49]	**0.40** [0.35, 0.44]
Sleep disturbance	**0.11** [0.06, 0.17]	**0.18** [0.12, 0.23]	**0.27** [0.22, 0.32]	**0.41** [0.36, 0.45]	0.29 [0.24, 0.34]
Social Participation	**-0.39** [-0.44, -0.35]	**-0.33** [-0.38, -0.29]	**-0.63 ** [-0.66, -0.60]	**-0.44** [-0.48, -0.39]	-0.24 [-0.29, -0.19]
Pain Interference	**0.44** [0.40, 0.48]	**0.41** [0.36, 0.45]	0.53 [0.49, 0.57]	**0.75 ** [0.72, 0.77]	**0.21** [0.15, 0.25]
Pain Intensity	**0.37** [0.33, 0.42]	**0.35** [0.30, 0.39]	**0.40** [0.35, 0.44]	**0.78** [0.76, 0.80]	0.19 [0.14, 0.24]

* Spearman’s rho coefficient was used to compare the T-scores of each PROMIS-29 domains (for pain intensity: numeric rating score) with each EQ-5D-5L item score. **Bold numbers** indicate confirmed hypotheses. [in brackets]: 95% confidence intervals for each correlation coefficient. The rationale behind these hypotheses is explained in Additional File 1

### Responsiveness

In total, 19 of 24 (79.2%) hypotheses were confirmed, including 10 out of 10 hypotheses for relative correlations, one out of five for pairs of *similar constructs*, three out of four for *related but dissimilar* constructs, and all five hypotheses targeting *unrelated constructs* (Table 5).

**Table 5:** Expected correlations between changes in scores of PROMIS-29 and EQ-5D-5L, and results

	Hypotheses addressing relative change scores (∆)	Confirmed Yes/No:
Expected relative correlations	PROMIS-29 Anxiety ∆ correlating higher with EQ-5D-5L Anxiety / Depression ∆ than with any other EQ-5D-5L dimension ∆	Yes
	PROMIS-29 Depression ∆ correlating higher with EQ-5D-5L Anxiety / Depression ∆ than with any other EQ-5D-5L dimension ∆	Yes
	PROMIS-29 Pain Interference ∆ correlating higher with EQ-5D-5L Pain / Discomfort ∆ than with any other EQ-5D-5L dimension ∆	Yes
	PROMIS-29 Pain Intensity ∆ correlating higher with EQ-5D-5L Pain / Discomfort ∆ than with any other EQ-5D-5L dimension ∆	Yes
	PROMIS-29 Social Participation ∆ correlating higher with EQ-5D-5L Usual Activities ∆ than with any other EQ-5D-5L dimension ∆	Yes
	PROMIS-29 Fatigue ∆ correlating higher with EQ-5D-5L Usual Care ∆ than with any other EQ-5D-5L dimension ∆	Yes
	PROMIS-29 Sleep Disturbance ∆ correlating higher with EQ-5D-5L Pain / Discomfort ∆ than with any other EQ-5D-5L dimension ∆.	Yes
	EQ-5D-5L Mobility ∆ correlating higher with PROMIS-29 Physical Function ∆ than with any other PROMIS-29 domain ∆	Yes
	EQ-5D-5L Self-Care ∆ correlating higher with PROMIS-29 Physical Function ∆ than with any other PROMIS-29 domain ∆	Yes
	EQ-5D-5L Usual Activities ∆ correlating higher with PROMIS-29 Physical Function ∆ than with any other PROMIS-29 domain ∆	Yes
Expected magnitude of the correlations	Hypotheses addressing change scores (∆) for the most similar constructs	Confirmed Yes/No*:
	PROMIS-29 Physical Function ∆ will correlate ≥ 0.5 with EQ-5D-5L Mobility ∆	No (0.49) [0.44, 0.54]
	PROMIS-29 Anxiety ∆ will correlate ≥ 0.5 with EQ-5D-5L Anxiety / Depression ∆	No (0.43) [0.37, 0.48]
	PROMIS-29 Depression ∆ will correlate ≥ 0.5 with EQ-5D-5L Anxiety / Depression ∆	No (0.33) [0.27, 0.38]
	PROMIS-29 Pain Interference ∆ will correlate ≥ 0.5 with EQ-5D-5L Pain / Discomfort ∆	No (0.42) [0.37, 0.47]
	PROMIS-29 Pain Intensity ∆ will correlate ≥ 0.5 with EQ-5D-5L Pain / Discomfort ∆	Yes (0.50) [0.45, 0.55]
	Hypotheses addressing change scores (∆) for related but dissimilar constructs	Confirmed Yes/No*:
	PROMIS-29 Physical Function ∆ will correlate in the interval between 0.30 and 0.50 with EQ-5D-5L Self-Care ∆	Yes (0.33) [0.27, 0.38]
	PROMIS-29 Physical Function ∆ will correlate in the interval between 0.30 and 0.50 with EQ-5D-5L Usual Activities ∆	Yes (0.45) [0.40, 0.49]
	PROMIS-29 Social Participation ∆ will correlate in the interval between 0.30 and 0.50 with EQ-5D-5L Usual Activities ∆	Yes (0.36) [0.30, 0.41]
	PROMIS-29 Sleep Disturbance ∆ will correlate in the interval between 0.30 and 0.50 with EQ-5D-5L Anxiety / Depression ∆	No (0.19) [0.13, 0.25]
	Hypotheses addressing change scores (∆) for the most unrelated constructs	Confirmed Yes/No*:
	PROMIS-29 Sleep Disturbance ∆ will correlate < 0.3 with EQ-5D-5L Mobility ∆	Yes (0.12) [0.06, 0.18]
	PROMIS-29 Anxiety ∆ will correlate < 0.30 with EQ-5D-5L Mobility ∆	Yes (0.04) [-0.02, 0.10]
	PROMIS-29 Depression ∆ will correlate < 0.30 with EQ-5D-5L Self-Care ∆	Yes (0.13) [0.07, 0.19]
	PROMIS-29 Fatigue ∆ will correlate < 0.30 with EQ-5D-5L Self-Care ∆	Yes (0.11) [0.20, 0.32]
	PROMIS-29 Sleep Disturbance ∆ will correlate < 0.30 with EQ-5D-5L Self-Care ∆	Yes (0.16) [0.10, 0.22]

*Spearman’s rho coefficient was used to compare changes in the T-scores of each PROMIS-29 domains (or numeric rating score for Pain Intensity) with changes in each EQ-5D-5L item score. [In brackets]: 95% confidence interval. The first 10 relative correlations are based on absolute numbers, and not tested for significant difference

## Discussion

In this study, the PROMIS-29 Profile v2.1 demonstrated sufficient internal consistency, construct validity, and responsiveness when used in a Norwegian rehabilitation context involving patients with a variety of chronic diseases. There were no floor effects in this heterogeneous sample of adults undergoing rehabilitation, though ceiling effects were observed in two domains.

### Floor and ceiling effects

The proportion of missing PROMIS-29 items was well below 5%, providing a comprehensive evaluation of the patients’ health status. We found no floor effects in this study, indicating no clustering at the lowest health scores across any PROMIS-29 domains. However, ceiling effects were present, with approximately one-fifth of the scores clustering at the best health levels in the Anxiety and Depression domains. At the item level, potential ceiling effects were also noted in Physical Function. The ceiling effects observed in our study were lower than those reported in previous research conducted in the general Norwegian population, with proportion reaching up to 54% at the best health end of scales for Anxiety and Depression [18, 19]. In those studies, pronounced clustering of scores at the best health end of scales were also observed in the domains of Physical Function, Fatigue, Social Participation, Pain Interference and Pain Intensity [18, 19]. The greater variability in responses to PROMIS-29 in our rehabilitation sample, compared to general population, strengthen the applicability of the questionnaire in clinical research and practice. While the presence of ceiling effects reduces the questionnaire’s responsiveness and precision at the healthier end of Anxiety and Depression scales, it still allows for the assessment of symptom deterioration or maintenance of symptom absence post-treatment. PROMIS Computer Adaptive Testing (CAT) can eliminate the ceiling effects in languages where the complete PROMIS Item Banks have been translated. PROMIS CAT is not yet available in Norway, however the PROMIS-57 profile or 8-item PROMIS short forms for anxiety and depression can similarly offer greater measurement precision in the healthier end of the scale. The T-score logic allows for interpreting scores from CAT, 4-item and 8-item PROMIS versions interchangeably and on the same scale.

### Internal consistency

The internal consistency for each PROMIS-29 domain was confirmed, with alpha and omega values both exceeding the COSMIN threshold of ≥ 0.70, and near or above 0.90 in all domains except Sleep disturbance. Our findings align with other studies that demonstrate sufficient internal consistency [17, 19, 38, 39] for this questionnaire.

### Construct validity

The construct validity of the PROMIS-29 was demonstrated by confirming 85.0% of the 40 predefined hypotheses, indicating that most domain scores followed the expected pattern, showing varying degrees of correlations with the dimensions from the EQ-5D-5L. All pairs of domain/dimension expected to represent the same constructs, or weakly / unrelated constructs, were confirmed. For the pairs that did not meet the threshold for large or moderate correlations, the correlation values were close, deviating no more than 0.09 from the expected levels. Our findings support the construct validity of the Norwegian version of PROMIS-29 in a rehabilitation setting, as previously demonstrated only in the Norwegian general population sample [18].

### Responsiveness

Responsiveness was demonstrated by confirming 79.2% of the predefined hypotheses. Although all change scores for relative correlations followed the expected pattern, some deviations were observed in the absolute correlations concerning pairs of items expected to represent the same constructs. The deviations may be explained by differences in the descriptive systems of the PROMIS-29 and EQ-5D-5L [34]. Despite conceptual overlaps, the more detailed items in the PROMIS-29 may not measure exactly the same as the more general statements in the EQ-5D-5L. However, the correlation values for the most similar constructs were close, deviating no more than 0.08 from expected levels. Our findings indicate that the PROMIS-29 can be used to measure changes in patient-reported HRQoL over time.

### Strengths and limitations

Strengths of this study include the COSMIN-based methodology [21, 30, 32, 40], the large study sample, and the use of the widely validated EQ-5D-5L as a comparator, with research evidence supporting the relationship between PROMIS-29 domains and EQ-5D-5L dimensions [18, 34, 35]. The use of digital data collection and the method of participant selection may have introduced sampling bias. Some of our findings may be less valid in immigrant populations, as the proportion of non-Scandinavian native tongue were higher among the participants who were lost to follow-up compared to our study sample. Limitations arising from differences in the questionnaires’ descriptive systems were addressed through hypothesized correlation levels and knowledge and clinical expertise of our research team. Some combinations of PROMIS-29 domains and EQ-5D-5L dimensions are conceptually very similar, supported both by face validity and previous studies, adding a degree of certainty that the change score correlations also would correlate strongly. Still, conceptually divergent combinations may be expected to be somewhat connected, based on clinical experience. Relying on the opinion of experienced practitioners and researchers when needed, may add a potential for biases hypotheses. The limited response options of EQ-5D-5L and the modest PROMIS-29 mean improvement achieved may have diminished the reliability of the change correlations. Another limitation was the overlap between some of the selected change correlation categories, resulting from reliance on team consensus. Adding more literature of relationship between the constructs embedded in the PROMIS-29 domains and the EQ-5D-5L dimensions could have strengthened the rationale for the hypotheses used in this study.

While the EQ-5D-5L may not be the optimal comparator for assessing responsiveness, as further exploration of its responsiveness is still needed [29], it was highly relevant in our study. Both PROMIS-29 and EQ-5D-5L are designed to measure patients’ self-perceived HRQoL. They cover key health dimensions, are generic in nature, and exhibit sufficient conceptual overlap, making the EQ-5D-5L a suitable comparator in this context.

### Implications

Our study was conducted in a new clinical setting compared to previous research on the Norwegian PROMIS-29, thereby providing new insights into measurement properties of this increasingly utilized questionnaire. Further, the use of PROMIS-29 in a longitudinal context also allowed assessment of responsiveness, which is a measurement property that has not yet been sufficiently explored.

The currently demonstrated measurement properties of PROMIS-29 support its use in clinical rehabilitation practice. Health professionals can confidently use PROMIS-29 to measure patients’ HRQoL, evaluate their progress over time, and tailor interventions based on each patient’s profile score. Researchers can include PROMIS-29 to measure rehabilitation outcomes and evaluate changes in HRQoL following interventions, and healthcare leaders can use PROMIS-29 data to inform development of rehabilitation services.

## Conclusion

In conclusion, this study provides evidence for the internal consistency, construct validity and responsiveness of the PROMIS-29 v2.1 in a Norwegian rehabilitation context involving patients with various chronic diseases. The use of the Norwegian version of PROMIS-29 v2.1 is recommended for both research and clinical practice targeting adults referred for rehabilitation services.

## Electronic supplementary material

Below is the link to the electronic supplementary material.

Supplementary Material 1

Supplementary Material 2

Supplementary Material 3

Supplementary Material 4

## Data Availability

Data supporting our findings are available from the authors upon reasonable request and with permission of the research board.

## Abbreviations

CAT: Computer Assisted Testing
COSMIN: Consensus-based Standards for the selection of health Measurement Instruments
EQ-5D-5L: EuroQol-5 Dimensions-5 Levels questionnaire
HRQoL: Health Related Quality of Life
IRT: Item Response Theory
PROM: Patient-Reported Outcomes Measures
PROMIS: Patient-Reported Outcomes Measurement Information System^®^
SD: Standard Deviation
T1: Time 1, admission to rehabilitation
T3: Time 3, 3-month follow up assessment
