# Supplementary material for: Measurement properties of the PROMIS-29 profile v2.1 in a Norwegian rehabilitation context
Source: J Patient Rep Outcomes. 2025 Jul 31;9:98. doi: 10.1186/s41687-025-00929-7 (PMC12314140; doi:10.1186/s41687-025-00929-7)
Supplement: Supplementary file 1 — Supplementary Material 1 [file 41687_2025_929_MOESM1_ESM.docx]

**Additional File 1:** Developing Hypotheses for the Construct Validity (at T1) of the PROMIS-29 v2.1

1. Consider the content and wording of the items and their overlap or divergence
2. Utilize evidence from previous studies that have examined the relationship between the two instruments

| General population in Norway  N=3200, mean age 51 years, 55 % female  Cross-sectional study | General population in Australia  N=3013, mean age 47 years, 51 % female  Cross-sectional study |
| --- | --- |
| Garratt AM, Coste J, Rouquette A, Valderas JM. The Norwegian PROMIS-29: psychometric validation in the general population for Norway. J Patient Rep Outcomes. 2021 Sep 9;5(1):86. doi: 10.1186/s41687-021-00357-3. PMID: 34499288; PMCID: PMC8427163. | Aghdaee M, Gu Y, Sinha K, Parkinson B, Sharma R, Cutler H. Mapping the Patient-Reported Outcomes Measurement Information System (PROMIS-29) to EQ-5D-5L. Pharmacoeconomics. 2023 Feb;41(2):187-198. doi: 10.1007/s40273-022-01157-3. Epub 2022 Nov 7. PMID: 36336773; PMCID: PMC9883346. |

1. Utilize the clinical and scientific expertise within the research group to discuss and negotiate until a consensus is reached on the expected degree of similarity.

|  | same construct | largely related but dissimilar constructs | moderately related but dissimilar constructs | weakly related or unrelated constructs |
| --- | --- | --- | --- | --- |
| Correlation value | ≥ 0.60 | < 0.60 and ≥ 0.30 | < 0.50 and ≥ 0.20 | < 0.30 |
| Chiarotto A, Terwee CB, Kamper SJ, Boers M, Ostelo RW. Evidence on the measurement properties of health-related quality of life instruments is largely missing in patients with low back pain: A systematic review. J Clin Epidemiol. 2018 Oct; 102:23-37. doi: 10.1016/j.jclinepi.2018.05.006. Epub 2018 May 21. PMID: 29793009. | | | | |

1. Consider potentially differences between general populations and the specialized rehabilitation cohort used in our study.
2. If needed: The results from the Norwegian study may be given more weights when considering the degree of correlations, compared to the results from the other study.

*Continues….*

*Continued…..* **Additional File 1:** Predefined Hypotheses with Rationales for Evaluating the Construct Validity of the PROMIS-29 v2.1

|  | **PROMIS-29 domain** | **EQ-5D-5L dimension** | **Results from Garratt el al., 2021** | **Results from Aghdaee et al., 2022** | **Expected degree of similarity** |
| --- | --- | --- | --- | --- | --- |
|  | Physical Function (current abilities) | Mobility (today) | 0.67 | 0.75 | The same construct  ≥ 0.60 |
| Item(s) | Are you able to do chores such as vacuuming or yard work? | I have [response options] problems in walking about |  |  |  |
|  | Are you able to go up and down stairs at a normal pace? |  |  |  |  |
|  | Are you able to go for a walk of at least 15 minutes? |  |  |  |  |
|  | Are you able to run errands and shop? |  |  |  |  |
| Response options | Without any difficulty, with a little / some / much difficulty, *or* unable to do | No / slight / moderate / severe problems *or* I am unable to walk about |  |  |  |
|  | Physical Function (current abilities) | Usual Activities (e.g. work, study, housework, family or leisure activities) (today) | 0.64 | 0.70 | The same construct  ≥ 0.60 |
| Item(s) | Are you able to do chores such as vacuuming or yard work? | I have [response options] problems doing my usual activities |  |  |  |
|  | Are you able to go up and down stairs at a normal pace? |  |  |  |  |
|  | Are you able to go for a walk of at least 15 minutes? |  |  |  |  |
|  | Are you able to run errands and shop? |  |  |  |  |
| Response options | Without any difficulty, with a little / some / much difficulty, *or* unable to do | No / slight / moderate / severe problems *or* I am unable to do my usual activities |  |  |  |
|  | Anxiety (in the past 7 days…) | Anxiety / Depression (today) | 0.73 | 0.72 | The same construct  ≥ 0.60 |
| Item(s) | I felt fearful | I am [response options] anxious or depressed |  |  |  |
|  | I found it hard to focus on anything other than my anxiety |  |  |  |  |
|  | My worries overwhelmed me |  |  |  |  |
|  | I felt uneasy |  |  |  |  |
| Response options | Never / rarely / sometimes / often / always | Not / slightly / moderately / severely / extremely |  |  |  |

**correlations measured by Sperman’s rho, absolute values without sign character;*

*Continued…..* **Additional File 1:** Predefined Hypotheses with Rationales for Evaluating the Construct Validity of the PROMIS-29 v2.1

|  | **PROMIS-29 domain** | **EQ-5D-5L dimension** | **Results from Garratt el al., 2021** | **Results from Aghdaee et al., 2022** | **Expected degree of similarity** |
| --- | --- | --- | --- | --- | --- |
|  | Depression (in the past 7 days…) | Anxiety / Depression (today) | 0.71 | 0.73 | The same construct  ≥ 0.60 |
| Item(s) | I felt worthless | I am [response options] anxious or depressed |  |  |  |
|  | I felt helpless |  |  |  |  |
|  | I felt depressed |  |  |  |  |
|  | I felt hopeless |  |  |  |  |
| Response options | Never / rarely / sometimes / often / always | Not / slightly / moderately / severely / extremely |  |  |  |
|  | Ability to Participate in Social Roles and Activities (current abilities) | Usual Activities (e.g. work, study, housework, family or leisure activities) (today) | 0.60 | 0.58 | The same construct  ≥ 0.60  (decision on “same” versus “largely related construct” was based on semantic considerations and clinical experiences) |
| Item(s) | I have trouble doing all of my regular leisure activities with others | I have [response options] problems doing my usual activities |  |  |  |
|  | I have trouble doing all of the family activities that I want to do |  |  |  |  |
|  | I have trouble doing all of my usual work (include work at home) |  |  |  |  |
|  | I have trouble doing all of the activities with friends that I want to do |  |  |  |  |
| Response options | Never / rarely / sometimes / usually / always | No / slight / moderate / severe problems *or* I am unable to do my usual activities |  |  |  |

**correlations measured by Sperman’s rho, absolute values without sign character;*

*Continues….*

*Continued…..* **Additional File 1:** Predefined Hypotheses with Rationales for Evaluating the Construct Validity of the PROMIS-29 v2.1

|  | **PROMIS-29 domain** | **EQ-5D-5L dimension** | **Results from Garratt el al., 2021** | **Results from Aghdaee et al., 2022** | **Expected degree of similarity** |
| --- | --- | --- | --- | --- | --- |
|  | Pain Inference (in the past 7 days…) | Pain / Discomfort (today) | 0.73 | 0.70 | The same construct  ≥ 0.60 |
| Item(s) | How much did pain interfere with your day to day activities? | I have [response options] pain or discomfort |  |  |  |
|  | How much did pain interfere with work around the home? |  |  |  |  |
|  | How much did pain interfere with your ability to participate in social activities? |  |  |  |  |
|  | How much did pain interfere with your household chores? |  |  |  |  |
| Response options | Never / rarely / sometimes / often / always | No / slight / moderate / severe extreme |  |  |  |
|  | Pain Intensity (in the past 7 days…) | Pain / Discomfort (today) | 0.79 | n.a | The same construct  ≥ 0.60 |
| Item(s) | How would you rate your pain on average? | I have [response options] pain or discomfort |  |  |  |
| Response options | 11-point numeric rating scale, from 0 (no pain) to 10 (worst imaginable pain) | No / slight / moderate / severe extreme |  |  |  |

**correlations measured by Sperman’s rho, absolute values without sign character; n.a = not available*

*Continues…*

*Continued…..* **Additional File 1:** Predefined Hypotheses with Rationales for Evaluating the Construct Validity of the PROMIS-29 v2.1

|  | **PROMIS-29 domain** | **EQ-5D-5L dimension** | **Results from Garratt el al., 2021** | **Results from Aghdaee et al., 2022** | **Expected degree of similarity** |
| --- | --- | --- | --- | --- | --- |
|  | Physical Function (current abilities) | Anxiety / Depression (today) | 0.29 | 0.27 | Weakly related or unrelated constructs  < 0.30 |
| Item(s) | Are you able to do chores such as vacuuming or yard work? | I am [response options] anxious or depressed |  |  |  |
|  | Are you able to go up and down stairs at a normal pace? |  |  |  |  |
|  | Are you able to go for a walk of at least 15 minutes? |  |  |  |  |
|  | Are you able to run errands and shop? |  |  |  |  |
| Response options | Without any difficulty, with a little / some / much difficulty, *or* unable to do | Not / slightly / moderately / severely / extremely |  |  |  |
|  | Anxiety (in the past 7 days…) | Mobility (today) | 0.21 | 0.19 | Weakly related or unrelated constructs  < 0.30 |
| Item(s) | I felt fearful | I have [response options] problems in walking about |  |  |  |
|  | I found it hard to focus on anything other than my anxiety |  |  |  |  |
|  | My worries overwhelmed me |  |  |  |  |
|  | I felt uneasy |  |  |  |  |
| Response options | Never / rarely / sometimes / often / always | No / slight / moderate / severe problems *or* I am unable to walk about |  |  |  |
|  | Anxiety (in the past 7 days…) | Self-Care (today) | 0.16 | 0.25 | Weakly related or unrelated constructs  < 0.30 |
| Item(s) | I felt fearful | I have [response options] problems washing or dressing my self |  |  |  |
|  | I found it hard to focus on anything other than my anxiety |  |  |  |  |
|  | My worries overwhelmed me |  |  |  |  |
|  | I felt uneasy |  |  |  |  |
| Response options | Never / rarely / sometimes / often / always | No / slight / moderate / severe problems *or* I am unable to wash and dress myself |  |  |  |

**correlations measured by Sperman’s rho, absolute values without sign character;*

*Continues…*

*Continued…..* **Additional File 1:** Predefined Hypotheses with Rationales for Evaluating the Construct Validity of the PROMIS-29 v2.1

|  | **PROMIS-29 domain** | **EQ-5D-5L dimension** | **Results from Garratt el al., 2021** | **Results from Aghdaee et al., 2022** | **Expected degree of similarity** |
| --- | --- | --- | --- | --- | --- |
|  | Sleep disturbance (in the past 7 days…) | Mobility (today) | 0.24 | 0.26 | Weakly related or unrelated constructs  < 0.30 |
| Item(s) | My sleep quality was… | I have [response options] problems in walking about |  |  |  |
|  | My sleep was refreshing |  |  |  |  |
|  | I had a problem with my sleep |  |  |  |  |
|  | I had difficulty falling asleep |  |  |  |  |
| Response options | Very poor /poor / fair / good / very good (sleep quality) *or* not at all / a little bit / somewhat / quite a bit / very much | No / slight / moderate / severe problems *or* I am unable to walk about |  |  |  |
|  | Sleep disturbance (in the past 7 days…) | Self-Care (today) | 0.19 | 0.23 | Weakly related or unrelated constructs  < 0.30 |
| Item(s) | My sleep quality was… | I have [response options] problems washing or dressing my self |  |  |  |
|  | My sleep was refreshing |  |  |  |  |
|  | I had a problem with my sleep |  |  |  |  |
|  | I had difficulty falling asleep |  |  |  |  |
| Response options | Very poor /poor / fair / good / very good (sleep quality) *or* not at all / a little bit / somewhat / quite a bit / very much | No / slight / moderate / severe problems *or* I am unable to wash and dress myself |  |  |  |

**correlations measured by Sperman’s rho, absolute values without sign character;*

*Continues…*

*Continued…..* **Additional File 1:** Predefined Hypotheses with Rationales for Evaluating the Construct Validity of the PROMIS-29 v2.1

|  | **PROMIS-29 domain** | **EQ-5D-5L dimension** | **Results from Garratt el al., 2021** | **Results from Aghdaee et al., 2022** | **Expected degree of similarity** |
| --- | --- | --- | --- | --- | --- |
|  | Physical Function (current abilities) | Self-Care (today) | 0.47 | 0.60 | Largely related but dissimilar  < 0.60 and ≥ 0.30 |
|  |  | Pain / Discomfort (today) | 0.54 | 0.58 | Largely related but dissimilar  < 0.60 and ≥ 0.30 |
|  | Fatigue (in the past 7 days…) | Usual Activities (e.g. work, study, housework, family or leisure activities) (today) | 0.48 | 0.43 | Largely related but dissimilar  < 0.60 and ≥ 0.30 |
| Item(s) | I felt fatigued | I have [response options] problems doing my usual activities |  |  |  |
|  | I have trouble starting things because I am tired |  |  |  |  |
|  | How run-down did you feel on average? |  |  |  |  |
|  | How fatigued were you on average? |  |  |  |  |
| Response options | not at all / a little bit / somewhat / quite a bit / very much | No / slight / moderate / severe problems *or* I am unable to do my usual activities |  |  |  |
|  | Fatigue (in the past 7 days…) | Pain / Discomfort (today) | 0.46 | 0.39 | Largely related but dissimilar  < 0.60 and ≥ 0.30 |
|  |  | Anxiety / Depression (today) | 0.53 | 0.58 | Largely related but dissimilar  < 0.60 and ≥ 0.30 |
|  | Sleep disturbance (in the past 7 days…) | Anxiety / Depression (today) | 0.41 | 0.45 | Largely related but dissimilar  < 0.60 and ≥ 0.30 |
|  | Ability to Participate in Social Roles and Activities (current abilities) | Mobility (today) | 0.48 | 0.49 | Largely related but dissimilar  < 0.60 and ≥ 0.30 |
|  |  | Pain / Discomfort (today) | 0.50 | 0.47 | Largely related but dissimilar  < 0.60 and ≥ 0.30 |
|  |  | Anxiety / Depression (today) | 0.47 | 0.51 | Largely related but dissimilar  < 0.60 and ≥ 0.30 |
|  | Pain Inference (in the past 7 days…) | Mobility (today) | 0.52 (**) | 0.63 | Largely related but dissimilar  < 0.60 and ≥ 0.30 |
|  |  | Usual Activities (e.g. work, study, housework, family or leisure activities) (today) | 0.55 (**) | 0.65 | Largely related but dissimilar  < 0.60 and ≥ 0.30 |

**correlations measured by Sperman’s rho, absolute values without sign character; (**) = most weight given to results from the Norwegian study*

*Continued…..* **Additional File 1:** Predefined Hypotheses with Rationales for Evaluating the Construct Validity of the PROMIS-29 v2.1

|  | **PROMIS-29 domain** | **EQ-5D-5L dimension** | **Results from Garratt el al., 2021** | **Results from Aghdaee et al., 2022** | **Expected degree of similarity** |
| --- | --- | --- | --- | --- | --- |
|  | Pain Intensity (in the past 7 days…) | Mobility (today) | 0.46 | n.a | Largely related but dissimilar  < 0.60 and ≥ 0.30 |
|  |  | Usual Activities (e.g. work, study, housework, family or leisure activities) (today) | 0.48 | n.a | Largely related but dissimilar  < 0.60 and ≥ 0.30 |

**correlations measured by Sperman’s rho, absolute values without sign character; n.a = not available*

*Continues…*

*Continued…..* **Additional File 1:** Predefined Hypotheses with Rationales for Evaluating the Construct Validity of the PROMIS-29 v2.1

|  | **PROMIS-29 domain** | **EQ-5D-5L dimension** | **Results from Garratt el al., 2021** | **Results from Aghdaee et al., 2022** | **Expected degree of similarity** |
| --- | --- | --- | --- | --- | --- |
|  | Anxiety (in the past 7 days…) | Usual Activities (e.g. work, study, housework, family or leisure activities) (today) | 0.31 | 0.31 | Moderately related but dissimilar, < 0.50 and ≥ 0.20 |
|  |  | Pain / Discomfort (today) | 0.30 | 0.27 | Moderately related but dissimilar, < 0.50 and ≥ 0.20 |
|  | Depression (in the past 7 days…) | Mobility (today) | 0.31 | 0.25 | Moderately related but dissimilar, < 0.50 and ≥ 0.20 |
|  |  | Self-Care (today) | 0.26 | 0.29 | Moderately related but dissimilar, < 0.50 and ≥ 0.20 |
|  |  | Usual Activities (e.g. work, study, housework, family or leisure activities) (today) | 0.42 | 0.34 | Moderately related but dissimilar, < 0.50 and ≥ 0.20 |
|  |  | Pain / Discomfort (today) | 0.35 | 0.28 | Moderately related but dissimilar, < 0.50 and ≥ 0.20 |
|  | Fatigue (in the past 7 days…) | Mobility (today) | 0.34 | 0.34 | Moderately related but dissimilar, < 0.50 and ≥ 0.20 |
|  |  | Self-Care (today) | 0.26 | 0.32 | Moderately related but dissimilar, < 0.50 and ≥ 0.20 |
|  | Sleep disturbance (in the past 7 days…) | Usual Activities (e.g. work, study, housework, family or leisure activities) (today) | 0.33 | 0.31 | Moderately related but dissimilar, < 0.50 and ≥ 0.20 |
|  |  | Pain / Discomfort (today) | 0.39 | 0.34 | Moderately related but dissimilar, < 0.50 and ≥ 0.20 |
|  | Ability to Participate in Social Roles and Activities (current abilities) | Self-Care (today) | 0.37 | 0.42 | Moderately related but dissimilar, < 0.50 and ≥ 0.20 |
|  | Pain Inference (in the past 7 days…) | Self-Care (today) | 0.35 (**) | 0.52 | Moderately related but dissimilar, < 0.50 and ≥ 0.20 |
|  |  | Anxiety / Depression (today) | 0.34 | 0.36 | Moderately related but dissimilar, < 0.50 and ≥ 0.20 |
|  | Pain Intensity (in the past 7 days…) | Self-Care (today) | 0.31 | n.a | Moderately related but dissimilar, < 0.50 and ≥ 0.20 |
|  |  | Anxiety / Depression (today) | 0.35 | n.a | Moderately related but dissimilar, < 0.50 and ≥ 0.20 |

**correlations measured by Sperman’s rho, absolute values without sign character; (**) = most weight given to results from the Norwegian study; n.a = not available*
