# Supplementary material for: Measurement properties of the PROMIS-29 profile v2.1 in a Norwegian rehabilitation context
Source: J Patient Rep Outcomes. 2025 Jul 31;9:98. doi: 10.1186/s41687-025-00929-7 (PMC12314140; doi:10.1186/s41687-025-00929-7)
Supplement: Supplementary file 2 — Supplementary Material 2 [file 41687_2025_929_MOESM2_ESM.docx]

**Additional File 2:** Plan for Developing the Hypotheses for the Responsiveness of the PROMIS-29 v2.1 between T1 and T3

**Hypotheses addressing change scores and the relative correlations:**

1. The results of validity testing in previous studies will be included as part of the rationale for the responsiveness hypotheses.

| General population in Norway, Cross-sectional study  N=3200, mean age 51 years, 55 % female | General population in Australia, Cross-sectional study  N=3013, mean age 47 years, 51 % female |
| --- | --- |
| Garratt AM, Coste J, Rouquette A, Valderas JM. The Norwegian PROMIS-29: psychometric validation in the general population for Norway. J Patient Rep Outcomes. 2021 Sep 9;5(1):86. doi: 10.1186/s41687-021-00357-3. PMID: 34499288; PMCID: PMC8427163. | Aghdaee M, Gu Y, Sinha K, Parkinson B, Sharma R, Cutler H. Mapping the Patient-Reported Outcomes Measurement Information System (PROMIS-29) to EQ-5D-5L. Pharmacoeconomics. 2023 Feb;41(2):187-198. doi: 10.1007/s40273-022-01157-3. Epub 2022 Nov 7. PMID: 36336773; PMCID: PMC9883346. |

1. For each row, identify the cell with the highest baseline correlation based on previous validity testing (Garratt et al 2021, Aghdaee et al 2023). The change (∆) in the PROMIS-29 domain for that cell is expected to have a stronger correlation with the ∆ in the EQ-5D-5L dimension for the same cell than any other cells in the same row.
2. For each column, identify the cell with the highest baseline correlation based on previous validity testing Garratt et al 2021, Aghdaee et al 2023). The ∆ in the EQ-5D-5L dimension for that cell is expected to have a stronger correlation with the ∆ in the PROMIS-29 domain for the same cell than any other cells in the same column.

| The PROMIS-29 v2.1 Profile, health domains: | EQ-5D-5L dimensions: | | | | | | | | | | | | | | |
| --- | --- | --- | --- | --- | --- | --- | --- | --- | --- | --- | --- | --- | --- | --- | --- |
|  | Mobility | | | Self-Care | | | Usual Activities | | | Pain / Discomfort | | | Anxiety / Depression | | |
| Physical Function | 0.67 | 0.75 |  | 0.47 | 0.60 |  | 0.64 | 0.70 |  | 0.54 | 0.58 |  | 0.29 | 0.27 |  |
| Anxiety | 0.19 | 0.21 |  | 0.16 | 0.25 |  | 0.31 | 0.31 |  | 0.30 | 0.27 |  | 0.73 | 0.72 |  |
| Depression | 0.31 | 0.25 |  | 0.26 | 0.29 |  | 0.42 | 0.34 |  | 0.35 | 0.28 |  | 0.71 | 0.73 |  |
| Fatigue | 0.34 | 0.34 |  | 0.26. | 0.32 |  | 0.48 | 0.43 |  | 0.46 | 0.39 |  | 0.53 | 0.58 |  |
| Sleep Disturbance | 0.24 | 0.26 |  | 0.19 | 0.23 |  | 0.33 | 0.31 |  | 0.39 | 0.34 |  | 0.41 | 0.45 |  |
| Ability to Participate in Social roles and Activities | 0.48 | 0.49 |  | 0.37 | 0.42 |  | 0.60 | 0.58 |  | 0.50 | 0.47 |  | 0.47 | 0.51 |  |
| Pain Interference | 0.52 | 0.63 |  | 0.35 | 0.52 |  | 0.55 | 0.65 |  | 0.73 | 0.70 |  | 0.34 | 0.36 |  |
| Pain Intensity | 0.46 | - |  | 0.31 | - |  | 0.48 | - |  | 0.79 | - |  | 0.35 | - |  |
|  | G et al  2021 | A et al 2023 | Rehab-nytte | G et al  2021 | A et al 2023 | Rehab-nytte | G et al  2021 | A et al 2023 | Rehab-nytte | G et al  2021 | A et al 2023 | Rehab-nytte | G et al  2021 | A et al 2023 | Rehab-nytte |

1. Ensure at least one hypothesis in each row and each column, respectively
2. To finalize the responsiveness hypotheses: Our preliminary responsiveness hypotheses may be adjusted based on the validity results from our study (RehabNytte). *This was done for two out of ten hypotheses addressing the relative correlations.*

*Continued…* **Additional File 2:** Developing Hypotheses for the Responsiveness of the PROMIS-29 v2.1 between T1 and T3

**Hypotheses addressing expected magnitude of the correlations regarding change scores**

1. Consider the possibility of lower change score correlations than baseline correlations.
2. Utilize the clinical and scientific expertise within the research group to discuss and negotiate until a consensus is reached on the expected degree of similarity.

|  | similar constructs | related but dissimilar constructs | unrelated constructs |
| --- | --- | --- | --- |
| Correlation value | ≥ 0.50 | < 0.50 and ≥ 0.30 | < 0.30 |
| Prinsen CAC, Mokkink LB, Bouter LM, Alonso J, Patrick DL, de Vet HCW, Terwee CB. COSMIN guideline for systematic reviews of patient-reported outcome measures. Qual Life Res. 2018 May;27(5):1147-1157. doi: 10.1007/s11136-018-1798-3. Epub 2018 Feb 12. PMID: 29435801; PMCID: PMC5891568. | | | |

1. The selection of hypotheses should reflect various parts of the mapping table;
2. Results from the Norwegian study (Garratt et al 2021) may be given most weights in selection of the most similar /related but dissimilar / unrelated constructs.
3. Establish the hypotheses, like this:

- “PROMIS-29 *Domain X* ∆ will correlate ≥ 0.50 with EQ-5D-5L *Dimension Y* ∆*”* for the most similar constructs.
- “PROMIS-29 *Domain X* ∆ will correlate in the interval between 0.30 and 0.50 with EQ-5D-5L *Dimension Y* ∆*”* for related but dissimilar constructs
- “PROMIS-29 *Domain X* ∆ will correlate < 0.30 with EQ-5D-5L *Dimension Y* ∆*”* for the most unrelated constructs
