# Supplementary material for: Measurement properties of the PROMIS-29 profile v2.1 in a Norwegian rehabilitation context
Source: J Patient Rep Outcomes. 2025 Jul 31;9:98. doi: 10.1186/s41687-025-00929-7 (PMC12314140; doi:10.1186/s41687-025-00929-7)
Supplement: Supplementary file 3 — Supplementary Material 3 [file 41687_2025_929_MOESM3_ESM.docx]

**Additional file 3**

**Table D** Proportion of missing, floor and ceiling effects* by item and domains of the PROMIS-29.

| **PROMIS-29 domain**  PROMIS-29 items | Missing (%) | Floor (%)  (worst health) | Ceiling (%)  (best health) |
| --- | --- | --- | --- |
| **Physical Function** | 0.2 | 4.8 | 7.4 |
| Are you able to do chores such as vacuuming or yard work? | 0.6 | 16.5 | 12.3 |
| Are you able to go up and down stairs at a normal pace? | 0.8 | 12.6 | 20.9 |
| Are you able to go for a walk of at least 15 minutes? | 0.7 | 12.4 | 32.5 |
| Are you able to run errands and shop? | 0.8 | 10.7 | 27.2 |
| **Anxiety** *In the past 7 days…* | 0.4 | 0.0 | **22.5** |
| I felt fearful. | 1.4 | 0.5 | 46.1 |
| I found it hard to focus on anything other than my anxiety. | 1.9 | 0.8 | 58.0 |
| My worries overwhelmed me | 1.7 | 0.5 | 39.0 |
| I felt uneasy. | 1.6 | 1.6 | 27.8 |
| **Depression** *In the past 7 days…* | 0.4 | 0.0 | **22.8** |
| I felt worthless. | 1.4 | 1.3 | 47.9 |
| I felt helpless. | 1.3 | 1.4 | 33.0 |
| I felt depressed. | 1.1 | 0.7 | 39.9 |
| I felt hopeless. | 1.4 | 1.0 | 50.7 |
| **Fatigue** *During the past 7 days…* | 0.4 | 3.3 | 3.7 |
| I feel fatigued. | 1.2 | 12.4 | 7.2 |
| I have trouble starting things because I am tired. | 1.4 | 8.5 | 9.7 |
| How run-down did you feel on average? | 1.8 | 6.9 | 8.0 |
| How fatigued were you on average? | 1.4 | 7.0 | 7.7 |
| **Sleep disturbance** *In the past 7 days…* | 0.4 | 4.3 | 1.1 |
| My sleep quality was... | 2.3 | 13.5 | 5.1 |
| My sleep was refreshing. | 2.2 | 20.0 | 3.0 |
| I had a problem with my sleep. | 1.4 | 10.0 | 14.1 |
| 2I had difficulty falling asleep. | 1.1 | 9.2 | 23.3 |
| **Ability to Participate in Social Roles and Activities** | 0.4 | 3.9 | 2.1 |
| I have trouble doing all of my regular leisure activities with others. | 1.0 | 9.3 | 8.3 |
| I have trouble doing all of the family activities that I want to do. | 1.3 | 7.1 | 8.0 |
| I have trouble doing all of my usual work (including work at home). | 1.0 | 11.7 | 5.6 |
| I have trouble doing all of the activities with friends that I want to do. | 1.4 | 10.4 | 4.0 |
| **Pain Interference** *In the past 7 days…* | 0.8 | 7.4 | 10.1 |
| How much did pain interfere with your day-to-day activities? | 1.0 | 11.6 | 11.7 |
| How much did pain interfere with work around the home? | 1.4 | 12.4 | 12.8 |
| How much did pain interfere with your ability to participate in social activities? | 1.1 | 12.9 | 16.8 |
| How much did pain interfere with your household chores? | 1.2 | 13.9 | 14.6 |
| **Pain Intensity** *In the past 7 days…*  *How would you rate your pain on average?*  *(numeric rating scale from 0 (no pain) to 10)* | 1.3 | 0.4 | 6.9 |
| **Percentage per domain raw scores at “4” or “20” and per item raw scores at “1” or “5”. Floor: worst health status, regardless of the scoring direction of the domain. Thus, low* Physical Function *and* Social Participation *scores, and high* Anxiety, Depression, Fatigue, Sleep Disturbance *and* Pain *scores were considered floor effects. Floor/ceiling effects at (≥15) at the domain level are marked with* ***bold numbers****.* | | | |

**Table E** Paired samples t-test for significance of improvement for PROMIS-29


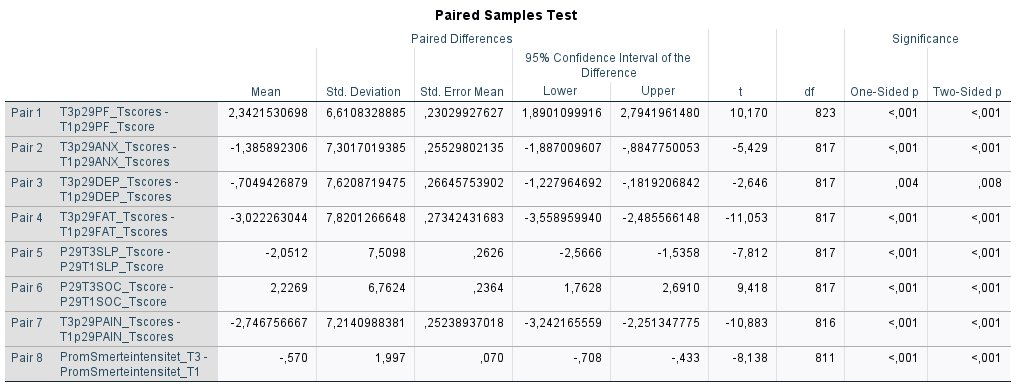


**Table F** PROMIS-29 Effect sizes of change

| **Paired Samples Effect Sizes** | | | | | | |
| --- | --- | --- | --- | --- | --- | --- |
|  | | | Standardizer^a^ | Point Estimate | 95% Confidence Interval | |
|  |  |  |  |  | Lower | Upper |
| Pair 1 | T3p29PF_Tscores - T1p29PF_Tscore | Cohen's d | 6,6108 | 0,354 | 0,284 | 0,425 |
|  |  | Hedges' correction | 6,6169 | 0,354 | 0,284 | 0,424 |
| Pair 2 | T3p29ANX_Tscores - T1p29ANX_Tscores | Cohen's d | 7,3017 | -0,190 | -0,259 | -0,121 |
|  |  | Hedges' correction | 7,3084 | -0,190 | -0,259 | -0,120 |
| Pair 3 | T3p29DEP_Tscores - T1p29DEP_Tscores | Cohen's d | 7,6209 | -0,093 | -0,161 | -0,024 |
|  |  | Hedges' correction | 7,6279 | -0,092 | -0,161 | -0,024 |
| Pair 4 | T3p29FAT_Tscores - T1p29FAT_Tscores | Cohen's d | 7,8201 | -0,386 | -0,457 | -0,315 |
|  |  | Hedges' correction | 7,8273 | -0,386 | -0,457 | -0,315 |
| Pair 5 | P29T3SLP_Tscore - P29T1SLP_Tscore | Cohen's d | 7,5098 | -0,273 | -0,343 | -0,203 |
|  |  | Hedges' correction | 7,5167 | -0,273 | -0,343 | -0,203 |
| Pair 6 | P29T3SOC_Tscore - P29T1SOC_Tscore | Cohen's d | 6,7624 | 0,329 | 0,259 | 0,400 |
|  |  | Hedges' correction | 6,7686 | 0,329 | 0,259 | 0,399 |
| Pair 7 | T3p29PAIN_Tscores - T1p29PAIN_Tscores | Cohen's d | 7,2141 | -0,381 | -0,452 | -0,310 |
|  |  | Hedges' correction | 7,2207 | -0,380 | -0,451 | -0,309 |
| Pair 8 | PromSmerteintensitet_T3 - PromSmerteintensitet_T1 | Cohen's d | 1,997 | -0,286 | -0,356 | -0,215 |
|  |  | Hedges' correction | 1,998 | -0,285 | -0,355 | -0,215 |

EQ-5D-5L Effect sizes of change

|  | | | Standardizer^a^ | Point Estimate | 95% Confidence Interval | |
| --- | --- | --- | --- | --- | --- | --- |
|  |  |  |  |  | Lower | Upper |
| Pair 1 | Eq5Dgange_T3 - Eq5Dgange_T1 | Cohen's d | 0,961 | -0,151 | -0,220 | -0,082 |
|  |  | Hedges' correction | 0,962 | -0,151 | -0,219 | -0,082 |
| Pair 2 | Eq5Dpersonligstell_T3 - Eq5Dpersonligstell_T1 | Cohen's d | 0,700 | -0,120 | -0,188 | -0,051 |
|  |  | Hedges' correction | 0,701 | -0,120 | -0,188 | -0,051 |
| Pair 3 | Eq5Dvanligegjøremål_T3 - Eq5Dvanligegjøremål_T1 | Cohen's d | 1,063 | -0,317 | -0,386 | -0,247 |
|  |  | Hedges' correction | 1,064 | -0,316 | -0,386 | -0,246 |
| Pair 4 | Eq5Dsmerterubehag_T3 - Eq5Dsmerterubehag_T1 | Cohen's d | 0,825 | -0,305 | -0,375 | -0,235 |
|  |  | Hedges' correction | 0,825 | -0,305 | -0,374 | -0,235 |
| Pair 5 | Eq5Dangstdepresjon_T3 - Eq5Dangstdepresjon_T1 | Cohen's d | 0,747 | -0,149 | -0,217 | -0,080 |
|  |  | Hedges' correction | 0,748 | -0,149 | -0,217 | -0,080 |
| Pair 6 | Eq5DVas_T3 - Eq5DVas_T1 | Cohen's d | 19,457 | 0,368 | 0,297 | 0,438 |
|  |  | Hedges' correction | 19,475 | 0,367 | 0,296 | 0,438 |

**Table F** Respondents % achieving more than Minimal Detectable Change, >3 T-score points improvement per PROMIS domain.

| PROMIS-29 domain  (min-max possible score range) | % of respondents reporting >3 T-score points improvement between T1 and T3 |  |  |
| --- | --- | --- | --- |
| Physical function | 33.8 |  |  |
| Anxiety | 34.8 |  |  |
| Depression | 32.2 |  |  |
| Fatigue | 45.2 |  |  |
| Sleep disturbance | 42.3 |  |  |
| Social Participation | 38.5 |  |  |
| Pain Interference | 40.2 |  |  |
|  | % of respondents achieving >2 NRS improvement |  |  |
| Pain Intensity (0-10) | 26.1 |  |  |
| *SD: standard deviation; T1: admission to rehabilitation; T3: three months after admission; NRS: numeric rating scale. Better health= positive T-score change for physical function and social participation, and negative change for all others.* | | |  |
