# Supplementary material for: Measurement properties of the PROMIS-29 profile v2.1 in a Norwegian rehabilitation context
Source: J Patient Rep Outcomes. 2025 Jul 31;9:98. doi: 10.1186/s41687-025-00929-7 (PMC12314140; doi:10.1186/s41687-025-00929-7)
Supplement: Supplementary file 4 — Supplementary Material 4 [file 41687_2025_929_MOESM4_ESM.docx]

**Additional file 4**

**Table H: Demographic and clinical variables, comparing the studied sample to the lost-to-follow-up**

| **Variables** | **Study sample, n=828** | **Lost sample, n=270** | **Significant difference  (p-value)** |
| --- | --- | --- | --- |
| Age^a^, years, mean (SD) | 54.3 (13.8) | 53.7 (13.7) | No (0.53) |
| Sex^a^, female, n (%) | 561 (67.8) | 175 (64.8) | No (0.61) |
| Diagnosis^b^, n (%) |  |  |  |
| Rheumatic or musculoskeletal diseases | 428 (51.7) | 137 (50.7) | No (0.85) |
| Neurological disease | 125 (15.1) | 38 (14.1) | No (0.72) |
| Lifestyle disease, overweight | 93 (11.2) | 28 (10.4) | No (0.72) |
| Cancer | 54 (6.5) | 19 (7.0) | No (0.76) |
| Sensory impairment | 44 (5.4) | 17 (6.3) | No (0.54) |
| Cardiovascular disease | 44 (5.4) | 14 (5.2) | No (0.95) |
| Mental disease | 4 (0.5) | 2 (0.7) | No (0.62) |
| Other disease | 36 (4.3) | 15 (5.6) | No (0.42) |
| Rehabilitation institution, n (%) |  |  |  |
| Centre 1 | 43 (5.2) | 22 (8.1) | No (0.09) |
| Centre 2 | 96 (11.6) | 28 (10.4) | No (0.61) |
| Centre 3 | 28 (3.4) | 8 (3.0) | No (0.77) |
| Centre 4 | 58 (7.0) | 13 (4.8) | No (0.21) |
| Centre 5 | 35 (4.2) | 23 (8.5) | **Yes (<0.001)** |
| Centre 6 | 153 (18.5) | 44 (16.3) | No (0.47) |
| Centre 7 | 94 (11.4) | 26 (9.6) | No (0.46) |
| Centre 8 | 13 (1.6) | 4 (1.5) | No (0.96) |
| Centre 9 | 16 (1.9) | 10 (3.7) | No (0.12) |
| Centre 10 | 65 (7.9) | 17 (6.3) | No (0.43) |
| Centre 11 | 11 (1.3) | 4 (1.5) | No (0.82) |
| Centre 12 | 23 (2.8) | 4 (1.5) | No (0.24) |
| Centre 13 | 44 (5.3) | 19 (7.0) | No (0.31) |
| Centre 14 | 104 (12.6) | 36 (13.3) | No (0.75) |
| Centre 15 | 45 (5.4) | 12 (4.4) | No (0.55) |
| **Patient-reported data** |  |  |  |
| Comorbidities^c^ n, median (min, max) | 2.4 (0, 10) | 2 (0, 9) | **Yes (<0.001)** |
| Body mass index kg/m^2^, mean (SD) | 30.1 (7.0) | 29.2 (6.9) | No (0.07) |
| Smoking and/or snuff use, n (%) | 201 (24.3) | 80 (29.6) | No (0.13) |
| Education >12 years, n (%) | 360 (43.5) | 111 (41.1) | No (0.61) |
| Paid work (currently, full or part time), n (%) | 445 (54.0) | 151 (55.9) | No (0.66) |
| Recipients of social security benefits, n (%) | 678 (81.9) | 218 (80.7) | No (0.86) |
| Language (native tongue), n (%) |  |  |  |
| Norwegian, Swedish, or Danish (Scandinavian) | 785 (94.8) | 246 (91.1) | No (0.59) |
| Other languages | 43 (5.2) | 24 (8.9) | **Yes (0.04)** |
| Civil status, n (%) |  |  |  |
| Married / cohabitant | 514 (62.1) | 162 (60.0) | No (0.71) |
| Single | 311 (37.6) | 108 (40.0) | No (0.57) |
| Caregiver for child(ren)/others in or outside home, n (%) | 352 (42.5) | 130 (48.1) | No (0.23) |
| Annual gross income in the household  >600 000 NOK, n (%) | 382 (46.0) | 119 (44.1) | No (0.67) |
| *^a^Data collected from the national identification number. ^b^Clinician-reported data, mandatory, ^c^Self-reported using a 19-item comorbidity checklist.* | |  |  |
